# Commentary on: “Motor Switching and Motor Adaptation Deficits Contribute to Freezing of Gait in Parkinson’s Disease”

**DOI:** 10.3389/fneur.2015.00084

**Published:** 2015-04-28

**Authors:** Yann Thibaudier

**Affiliations:** ^1^Département de physiologie et biophysique, Université de Sherbrooke, Sherbooke, QC, Canada

**Keywords:** freezing of gait, split-belt treadmill, locomotion, Parkinson’s disease, coordination

More than 50% of Parkinson’s disease (PD) patients exhibit freezing of gait (FoG) characterized by a sudden and transient inability to initiate or continue walking (1). The pathophysiology of FoG is still unknown, particularly due to the difficulty in reproducing this symptom spontaneously in the laboratory, either in humans or in animal models (2, 3). A current hypothesis proposes that FoG could be caused by an asymmetry and impaired left-right coordination of the locomotor pattern (4).

In a recent article published in *Neurorehabilitation and Neural Repair*, Mohammadi and colleagues used a split-belt treadmill that can independently control the speed of the left and right sides to evaluate gait symmetry and adaptation in healthy subjects (*n* = 12), non-freezer (*n* = 12), and freezer (*n* = 10) PD patients (5). Subjects were evaluated in six conditions without interruption (each lasting 2 min): (1) slow tied-belt condition (3 km/h), (2) split-belt walking following an increase in speed of one side in a random manner to 4 km/h, (3) a return to slow tied-belt condition, (4) same split-belt condition for the other leg, (5) another return to slow tied-belt, and (6) finally, a fast tied-belt condition (4 km/h).

The results show that during the first tied-belt condition, freezer patients already exhibited more asymmetrical gait than non-freezer patients and healthy subjects. In addition, the changes of asymmetry during both switching to split-belt and returning to tied-belt were significantly larger in freezers compared to non-freezers and control subjects. Moreover, the adaptation of gait asymmetry during split-belt and the re-adaptation during return to tied-belt were slower in freezers. However, the most interesting result of this study is probably the fact that immediately after switching to split-belt with the most affected leg on the faster belt, one patient experienced a FoG episode and another one exhibited a festination episode. As a result, Mohammadi et al. (5) proposed that switching to an asymmetrical walking could be a primary deficit in freezers.

As stated in the introduction, it is difficult to evaluate FoG in laboratories, either in humans or in animal models (2, 3). Indeed, in a research environment, even patients with clear FoG fail to express this phenomenon during experimental testing (3). To better understand the pathophysiology of this symptom, some methods have been proposed to elicit FoG during standardized conditions. Snijders et al. (3) evaluated the effect of obstacle avoidance during treadmill walking on FoG. Of 13 PD patients, 8 exhibited FoG episodes during treadmill walking with obstacle avoidance. However, FoG episodes elicited by obstacle avoidance were brief and it was proposed that obstacle could act as a cue to rapidly help abort the episode. More recently, the same group investigated the percentage of FoG’s occurrence during different tasks such as normal walking, rapid short steps, or turning (6). Although rapid short steps represented an interesting method to provoke FoG in PD patients; this phenomenon was more frequent and duration of episodes was longer during turning task. However, standardized conditions are not often used for this kind of task and it remains difficult to compare the results between studies and research groups. As presented here, motor switching to split-belt locomotion can elicit FoG episodes and could represent an alternative method to study this symptom systematically in both PD patients and animal models of PD. Indeed, it was suggested that split-belt locomotion can simulate a turning task. In this case, FoG during switching to split-belt locomotion could be a more reliable phenomenon that appears during turning on overground locomotion. Only one patient exhibited FoG and another festination episode in Mohammadi’s study; however, just one difference of speed was used and the acceleration was not defined. In order to systematically induce FoG episodes by transition to split-belt locomotion, it will be necessary to refine the different parameters and evaluate if split-belt treadmill can be used to investigate FoG in laboratories.

The data presented by Mohammadi et al. (5) also support the hypothesis that FoG could be associated with an impaired coordination, particularly when the task requires asymmetric demands. Plotnik and Hausdorff (4) evaluated the phase coordination index (PCI) in patients with or without FoG during overground locomotion. Patients with FoG had a higher PCI corresponding to a lower level of coordination than those without FoG. Moreover, this decrease of coordination in patients with FoG became particularly important during turning tasks (7). Frazzitta et al. (8) also observed an impaired left–right symmetry during treadmill walking in PD patients, with a more pronounced tendency for freezers compared to non-freezers. These patients received treadmill training for 30 min every day, 5 days/week for 4 weeks. During this training, visual and auditory cues were provided to the patient to improve left-right symmetry. Training reduced the asymmetry and the frequency of FoG. In the same direction, Fasano et al. (9) evaluated gait asymmetry and FoG in patients implanted bilaterally for deep brain stimulation (DBS) of the subthalamic nucleus. They showed that reducing stimulation intensity of the side contralateral to the leg with the longest step decreased the frequency and duration of FoG episodes by an improvement of left-right symmetry and coordination. These studies showed that therapeutic strategies aimed at improving left-right symmetry in PD patients could reduce the occurrence and duration of FoG episodes. In a recent paper, Reisman et al. (10) investigated split-belt treadmill training to reduce step length asymmetry in post-stroke patients (10). Training was performed during 4 weeks with speeds chosen in order to exaggerate left-right asymmetry during split-belt locomotion. Of the 12 participants evaluated, 7 had a significant improvement in step length asymmetry after the training as well as 1 and 3 months later. In regards to the potential role of left-right asymmetry in FoG, new therapeutic strategies of split-belt treadmill training designed to reduce left-right asymmetry and to increase switching performance to an asymmetrical condition could be considered to treat FoG in PD patients.

## Conflict of Interest Statement

The author declares that the research was conducted in the absence of any commercial or financial relationships that could be construed as a potential conflict of interest.
